# Extraction of Proteins and Other Intracellular Bioactive Compounds From Baker’s Yeasts by Pulsed Electric Field Treatment

**DOI:** 10.3389/fbioe.2020.552335

**Published:** 2020-12-15

**Authors:** Valentina Ganeva, Boyana Angelova, Bojidar Galutzov, Vasilij Goltsev, Miroslava Zhiponova

**Affiliations:** Biological Faculty, Sofia University “St. Kl. Ohridski”, Sofia, Bulgaria

**Keywords:** pulsed electric field treatment, flow system, baker’s yeast, extraction, protein, bioactive compounds

## Abstract

Yeasts are rich source of proteins, antioxidants, vitamins, and other bioactive compounds. The main drawback in their utilization as valuable ingredients in functional foods and dietary supplements production is the thick, indigestible cell wall, as well as the high nucleic acid content. In this study, we evaluated the feasibility of pulsed electric field (PEF) treatment as an alternative method for extraction of proteins and other bioactive intracellular compounds from yeasts. Baker’s yeast water suspensions with different concentration (12.5–85 g dry cell weight per liter) were treated with monopolar rectangular pulses using a continuous flow system. The PEF energy required to achieve irreversible electropermeabilization was significantly reduced with the increase of the biomass concentration. Upon incubation of the permeabilized cells in water, only relatively small intracellular compounds were released. Release of 90% of the free amino acids and low molecular UV absorbing compounds, 80% of the glutathione, and ∼40% of the total phenol content was achieved about 2 h after pulsation and incubation of the suspensions at room temperature. At these conditions, the macromolecules (proteins and nucleic acids) were retained largely inside. Efficient protein release (∼90% from the total soluble protein) occurred only after dilution and incubation of the permeabilized cells in buffer with pH 8–9. Protein concentrates obtained by ultrafiltration (10 kDa cut off) had lower nucleic acid content (protein/nucleic acid ratio ∼100/4.5) in comparison with cell lysates obtained by mechanical disintegration. The obtained results allowed to conclude that PEF treatment can be used as an efficient alternative approach for production of yeast extracts with different composition, suitable for application in food, cosmetics and pharmaceutical industries.

## Introduction

Baker’s yeast is currently the most distributed commercial yeast worldwide. It consists of one or several selected strains of *Saccharomyces cerevisiae*, which are able to develop very fast and give high biomass quantities. The primary raw material utilized for their cultivation is molasses, a by-product of the refining of sugar beets and sugar cane, which makes their production on industrial scale very cost effective (Pérez-Torrado et al., 2015).

Besides its principal use as a leavening agent in baking and in other traditional fermentation processes, such as wine and beer making, *S. cerevisiae* which has GRAS (Generally recognized for safe) status has found a variety of applications in agriculture, food, cosmetics, and pharmaceutical industries (Pérez-Torrado et al., 2015; Żymańczyk-Duda et al., 2017).

Yeast are a rich source of proteins with a high level of many of the essential amino acids. *S. cerevisiae* is among the most widely used microorganisms for production of Single Cell Protein for animal feed and human diet (Halász and Lásztity, 1991; Bekatorou et al., 2006). Due to its high content of vitamins, minerals, antioxidants, and other bioactive compounds, the yeast biomass is used in different preparations as health supplements and natural flavor compounds for food industry and as additives for the cosmetic industry (Abbas, 2006).

Today, *S. cerevisiae* is one of the main microorganisms for fermentative production of glutathione for food and pharmaceutical industries (Li et al., 2004). The yeast is also an important source of β-glucans, many industrial enzymes and metabolites (Żymańczyk-Duda et al., 2017).

The yeast biomass can be used in different ways—as whole cells after inactivation by heating (inactive dry yeast), as autolysates and hydrolysates, or after extraction, fractionation, and purification of the valuable intracellular compounds (Halász and Lásztity, 1991; Bekatorou et al., 2006).

*S. cerevisiae*, in the form of inactivated whole cells, is used nowadays mainly as a protein supplement in animal feeding. Despite the high content of protein and other bioactive substances, there are two main factors, which restrict the use of whole cells in a human diet. The yeast has a very rigid, indigestible cell wall, which decreases the bioavailability of the intracellular compounds and can provoke allergies and gastrointestinal disorders (Kinsella and Shetty, 1978). The other serious limitation is the high nucleic acid (NA) content, which for yeast is in the range of 10–15% on dry cell bases (Halász and Lásztity, 1991; Nasseri et al., 2011). Human body lacks the enzyme uricase meaning that purine metabolism leads to the production of uric acid in humans. Overproduction of uric acid plays an emerging role in many human diseases (Maiuolo et al., 2016). Therefore, the utilization of yeast protein for human consumption requires destruction of the cell wall and significant reduction of the NA contents.

*S. cerevisiae* is the main starting material for production of yeast extracts with different composition. Currently at the industrial level there are two main methods for production of yeast extracts: (a) by autolysis, i.e. digestion of the cell wall components and the intracellular macromolecules by endogenous enzymes, a process which can be further optimized by addition of exogenous enzymes and chemical treatment; (b) by mechanical disintegration (Halász and Lásztity, 1991; Nasseri et al., 2011; Jacob et al., 2019). Yeast autolysates are used mainly as a component of microbial culture media while in the food industry they are added as a flavoring agent in many foods at 0.5–2% content. Mechanical disintegration is applied as a first step for production of polysaccharides (β-glucan) from yeast and is also the main method for extraction of proteins, antioxidants and other soluble intracellular products from yeast (Middelberg, 2012; Bzduhcha-Wróbel et al., 2014). Despite being routinely applied in biotechnology, mechanical disintegration leads to non-selective release of the product, increase in the viscosity, and micronization of the cell debris, which complicates the liquid–solid separation and the following downstream processing (Balasundaram et al., 2009). On the other hand, when the yeast derived compounds are intended for use in the food, cosmetics or pharmaceutical industry, multiple steps of purification are needed in order to obtain an odorless, tasteless, and colorless product which furthermore does not contain potentially harmful substances. In this case, a more selective and mild extraction procedure can simplify the production process and make it less expensive and time consuming.

During the last few years many data have been accumulated on the application of pulsed electric field (PEF) treatment as an alternative, non-thermal method for extraction of bioactive compounds from microorganisms and plant tissues (Lebovka et al., 2005; Golberg et al., 2016; Postma et al., 2016). The electrical treatment provokes plasma membrane permeabilization known as electropermeabilization or electroporation, which is associated with the induction of an additional transmembrane potential (Rols and Teissie, 1990; Weaver and Chizmadzhev, 1996). Depending on the electrical parameters, the cell characteristics (size, age, and shape), the pulsing media composition etc., the loss of the membrane integrity can be reversible or irreversible. The electropermeabilization is associated with a leakage of many soluble intracellular compounds. The extraction efficiency depends on the fraction of the irreversibly permeabilized cells and in case of macromolecules such as proteins and NA can vary greatly in dependence of the cell wall structure and the conditions of the post-pulse incubation (Ganeva et al., 2003; Shynkaryk et al., 2009; Liu et al., 2012; Goettel et al., 2013; Coustets et al., 2015).

It has been shown recently, that irreversible electropermeabilization by using a continuous flow system followed by an incubation of the cells in buffer with a pH 7–7.5 led to massive release of native intracellular enzymes from different yeast species (Ganeva et al., 2003, 2014). At these conditions, a release of about 40–50% from the total soluble protein was observed, depending on the species and cell growth phase.

In the present study, we evaluated the feasibility of PEF treatment applied in a flow mode as an alternative method for extraction of protein and low molecular weight biologically active compounds from fresh baker’s yeast.

## Materials and Methods

### Materials

2,2′-bis(3-ethylbenzthiazoline-6-sulfonic acid) diammonium salt (ABTS) and dithiothreitol (DTT) were purchased from GERBU (Germany). Propidium iodide (PI), lyticase, reduced L-glutathione and glass beads were purchased from Sigma-Aldrich (Germany), other chemicals were bought from AppliChem (Germany).

### Yeast Strain

The experiments were performed with commercial fresh baker’s yeast (VIVO, Lesaffre Magyarország). The cells were washed once, resuspended in distilled water and incubated for 1 h. Next, the suspension was centrifuged, and the cells were diluted in distilled water to final concentrations corresponding to 12.5–85 g dry cell weight per liter (gDCW L^–1^). The suspensions conductivity was adjusted to 150 ± 5 μS cm^–1^ with 0.2 M KCl.

### Pulsed Electric Field (PEF) Treatment

The electric field treatment in a continuous-flow chamber was performed with a generator of monopolar rectangular pulses (2300V-10A), a Hydropuls mini specially fabricated GBS-Elektronik (Germany). The pulse duration and frequency were regulated by an arbitrary waveform generator RIGOL DG1012 (China). The chambers (0.3 and 0.5 mL volume) have two parallel stainless steel electrodes that are 0.3 and, respectively, 0.4 cm apart, specially manufactured by MEHEL (Bulgaria). The PEF treatment was performed at flow rates of 9, 27, and 130 mL min^–1^, controlled by a peristaltic pump. During the passage through the chamber, the cells received a train of pulses with a duration of 0.8 or 0.5 ms and electric field strength range of 2.5–5.5 kV cm^–1^ (Table 1). The total treatment time (*t*) was defined as the number of pulses (n) multiplied by the single pulse duration (τ):

**TABLE 1:** PEF treatment conditions.

Pulsing chamber (mL)	Flow rate (mL min^–1^)	Pulse number	Pulse duration (ms)	Pulse freqency (Hz)	Cell concentration (gDCW L^–1^)
0.3	9	15	0.8	7.5	12.5–85
0.5	27	19	0.5	17.1	66
	130	19	0.5	82.3	66

(1) t=n⋅τ

All pulsing parameters were monitored online with an Instek GDS 2064 oscilloscope (Taiwan). Schematic representation of the experimental setup used for PEF treatment is included as Supplementary Material 1.

The specific treatment energy per liter of cell suspension (W_sus_, kJ L^–1^) was then calculated by dividing the average power associated to the train of pulses (P_avg_, W) by the flow rate [Φ (L s^–1^)] (Lindgren et al., 2002):

(2) $$W_{s\,u\,s}=\frac{P_{a\,v\,g}}{\Phi }=\frac{U\cdot I\cdot \tau \cdot f}{\Phi }$$

where U is the applied voltage (V), I is the current (A), τ is the single pulse duration (s), and f is the pulse frequency (Hz).

The inlet temperature of the suspensions was 23–24°C. The outlet temperature was registered by a K-type thermocouple connected to a digital thermometer, and the sensor was attached to the end of the tubing. After pulsation, the suspensions were incubated at room temperature or alternatively were diluted in potassium phosphate buffer (PPB) to final concentration of 167 mM and incubated at room temperature. Samples were taken at various times after pulsation and centrifuged for 2 min at 10,750×*g* in an Eppendorf centrifuge or for 10 min at 2,000×*g* (ROTINA 380). The supernatants were kept at 4°C until further analysis.

### Determination of Irreversible Electropermeabilization

The membrane permeabilization was assayed by loading the cells with PI. To determine the fraction of cells with irreversibly permeabilized membranes, 5 μL of 0.5 mM solution of PI in distilled water were added to 50 μL cell suspension 1 h after pulsation of the cells. The number of fluorescent cells was counted under an epifluorescent microscope (L3201 LED, Microscopesmall, China). The permeabilization was expressed as a percentage of the number of fluorescent cells relative to the total cell number.

### Preparation of the Cell Lysate

At first, 1 mL water suspension of control, untreated cell was mixed with an equal volume of glass beads (diameter 0.42–0.6 mm, Sigma) and vortexed 8 times for 1 min each, with 15 s pause intervals with ice incubation, respectively. The resulting lysate was centrifuged at 10,750×*g* for 5 min, and the supernatant was kept at 4°C until used for amino acid, total antioxidant activity, glutathione and total phenol content analyses.

Secondly, control yeast cells diluted in PPB to the same concentration as electrically treated cells, were mixed with an equal volume of glass beads and vortexed 8 times for 1 min each, with 30 s pause intervals with ice incubation, respectively.

This procedure led to about 99% cell lysis, as determined by counting the cells in a Thoma chamber.

### Lyticase Test

To detect electroinduced changes in the cell wall porosity, pulsed and untreated, control cell suspensions were diluted in PPB pH 7.5 (167 mM final concentration) to 10 mgDCW mL^–1^. To this suspension, lyticase was added to final concentration of 60 U mL^–1^ and the suspensions were incubated at 30°C. At different intervals, samples were added to 1,000 μL of distilled water, and the optical density (OD) at 660 nm was determined. A decrease of the OD directly reflects the degree of cell lysis. OD values measured at different intervals after enzyme addition were expressed as percentages and were plotted against time. The OD of the sample before enzyme addition was 100%.

### Determination of the Release of UV Absorbing Substances From Electropermeabilized Cells

Cell suspensions (66 gDCW L^–1^) were treated at a flow rate of 27 mL min^–1^ in pulsing chamber with 0.5 mL volume with 19 pulses of 0.5 ms duration (total treatment time of 9.5 ms). The field strength was set at 3 kV cm^–1^. Samples of 0.5 mL were taken at various times after pulsation and centrifuged for 2 min at 10,750×*g* in an Eppendorf centrifuge. Next, 20 μL of the supernatants were mixed with 980 μL distilled water and the absorbance at 260 nm was read by using UV/VIS spectrophometer BOECO.

### Analytical Methods

Total protein content was determined according to Bradford (1976) with bovine serum albumin as a standard. Free amino acids were determined with Ninhydrin method according to Lamoolphak et al. (2006) with leucine as a standard, the total purine content was determined according to Loring et al. (1952) and the NA according to Spirin (1958). The alcohol dehydrogenase activity (ADH) was determined according to Scopes et al. (1981).

Total antioxidant activity was determined by TEAC (Trolox equivalent antioxidant capacity) assay as described by Pellegrini et al. (2003) with slight modifications. The ABTS**^+^** stock solution (7 mM aqueous solution of ABTS with 2.45 mM potassium persulfate) was diluted with 50 mM PPB, pH 7 to the absorbance of 0.70 ± 0.02 at 734 nm. The samples (10 μL) of fivefold diluted water extracts were mixed with 990 μL of working solution. The decrease of the absorbance at 734 nm was measured after 15 min. The Trolox standard curve was determined in the range of 0.1–15 μM. The data are expressed as mg Trolox Equivalent per gram of cell dry weight—mgTE gDCW^–1^.

The reduced form of glutathione (GSH) was determined by the colorimetric method with 5,5′-dithiobis-(2-nitrobenzoic acid), DTNB according to Rahman et al. (2006) with modifications. Briefly, 20 μL of 0.4% solution of DTNB in 0.1 M PPB pH 7.5, 5 mM EDTA were added to 960 μL of the same buffer. Afterward, 20 μL samples (supernatant of electrically treated and control cell suspensions, soluble fraction of the cell lysate obtained after mechanical disintegration) were added and the suspensions were incubated for 2–10 min at room temperature. The absorbance at 412 nm in triplicates was measured and the concentration of GSH was obtained from a standard curve using reduced L-glutathione.

The total phenolic content was determined according to Singleton et al. (1999) with modifications. Test samples contained 0.1 mL supernatant of electrically treated and control cell suspensions or cell lysate, 1.5 mL Folin-Ciocalteau reagent (previously diluted 10 times in distilled water), 1.4 mL 7.5% Na_2_CO_3_. The samples were incubated at dark, at room temperature for 30 min. The absorbance was measured at 765 nm by spectrophotometer Shimadzu UV 1800. Standard curve, made with known concentrations of gallic acid (GA), was used to calculate the quantity of phenolic compounds as gallic acid equivalents per fresh weight (mg gallic acid g^–1^ FW).

The yeast biomass was measured as dry weight using an infrared moisture analyzer method according to Li and Mira de Orduña (2010).

### Statistics

The results from three to six independent experiments are shown as a mean value ± SD. The Student *t*-test was applied for statistical evaluation. Differences were considered significant if *P* < 0.05.

## Results and Discussion

### Electroinduced Extraction of Protein

#### Influence of Medium pH on the Protein Release Efficiency

As shown earlier with other yeast systems, significant release of intracellular proteins occurs only under electrical conditions leading to irreversible plasma membrane damage, i.e., irreversible electropermeabilization (Ganeva et al., 2003, 2015).

In this study, to obtain a suitable combination of electrical parameters, the suspensions with a biomass concentration corresponding to 37.5 gDCW L^–1^ were subjected to 15 pulses of 0.8 ms duration with 7.5 Hz frequency at a flow rate of 9 mL min^–1^. The electrical treatment was performed in a continuous flow chamber with a volume of 0.3 mL. Optimization of the electropulsation protocol was performed by varying the field strength in the range of 2–4 kV cm^–1^. The degree of irreversible permeabilization was determined by loading the cells with PI.

We found that irreversible permeabilization of 99–100% of the cells is reached at 3.3 kV cm^–1^. Therefore, to evaluate the effect of the pH on the protein release efficiency, the cells were permeabilized at this field strength. After electrical treatment, the cell suspensions were diluted in potassium phosphate buffer (PPB) with pH 5–9 to final cell concentration of 12.5 gDCW L^–1^ (final buffer concentration 167 mM). DTT was added to a final concentration of 2 mM and the suspensions were incubated for 16 h at room temperature (24°C). Next, the cells were removed by centrifugation and the protein concentration in the supernatant of pulsed cells was determined as described in section “Materials and Methods.”

According to Sergeev et al. (1984), the pH of the medium has a significant impact on the protein extractability from mechanically disintegrated yeast cells. In their study, the lowest extraction efficiency (30% from total) was observed at pH 4–5, while maximal yield was reached in a medium with a pH 8.5–12 when the yield increase reaches an approximate plateau above pH 9.

In our hands, maximal yield of soluble protein after mechanical disintegration of the cells was reached at pH 8.5–8.75. Therefore, in order to estimate the protein release efficiency, we set as 100% the protein released from cells disintegrated in PPB with pH 8.5 containing 2 mM DTT which was 199 ± 8 mg gDCW^–1^.

**FIGURE 1:**
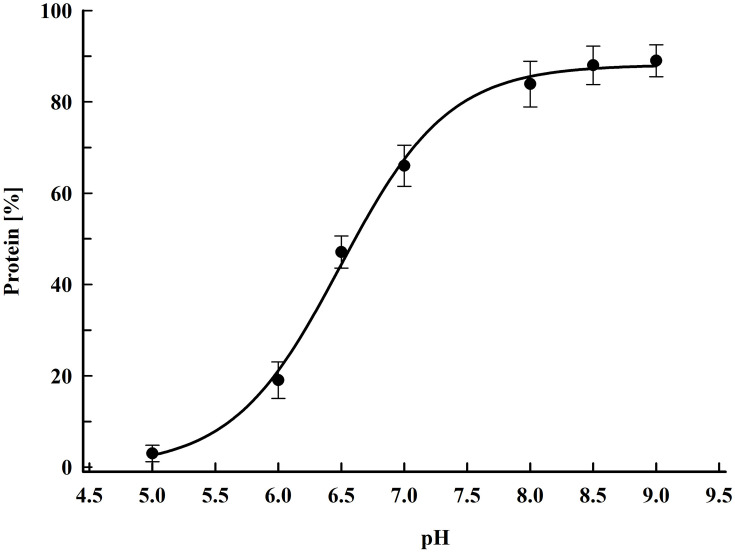
Influence of pH on protein release efficiency from electropermeabilized cells. Electrical conditions: field strength 3.3 kV cm^–1^, 15 pulses of 0.8 ms duration, pulse frequency 7.5 Hz, flow rate 9 mL min^–1^. After PEF treatment, the cells were diluted in PPB, containing 2 mM DTT and incubated for 16 h at room temperature. 100% corresponds to the total protein in the cell lysate. The values represent the mean ± SD of five different experiments.

As shown in Figure 1, more substantial protein release occurred at pH above 6. The maximal yield (88 ± 9%), was obtained by incubation in a buffer with pH 8.5–9. The protein release from permeabilized cells incubated in water was about 2% from the total protein. The protein release from control cells incubated for 16 h in 167 mM PPB pH 8.5 with 2 mM DTT was 1.75 ± 0.2%. Taking into account these results, we concluded, that it is rather the pH then the ionic strength, which so strongly affects the efflux of intracellular proteins.

In yeast, the cytoplasmic buffering capacity is determined mainly by inorganic phosphate (Poznanski et al., 2013). The electroinduced loss of plasma membrane integrity results in a very fast release of ions into the medium (Ganeva et al., 2003; Pavlin et al., 2007). Here we confirmed that after electrical treatment, leading to irreversible permeabilization, the conductivity of undiluted yeast suspension reached stable value of 1.5 ± 0.2 mS cm^–1^ within 10 min. The pH of cell suspension with the same concentration was 4.3–4.5 before treatment, and within 10–15 min after treatment reached values of about 5.5.

The isoelectric point for most of the cytoplasmic proteins in yeast is in the range of 4.5–6 (Schwartz et al., 2001). The cytoplasmic pH varies depending on the growth phase and nutrient starvation. For cells in exponential growth phase, cytoplasmic pH is 7–7.2 (Valli et al., 2005; Orij et al., 2009). The yeast cells respond to nutrient starvation by arresting growth and entering stationary phase, which is associated with many morphological and physiological changes. One of them is the decrease of cytoplasmic pH to 5.7–5.8 (Orij et al., 2009). Recent evidence showed that this acidification led to interaction of many soluble proteins with each other and reversible formation of assemblies and filaments, which triggers the transition of the cytoplasm from fluid—to a solid-like state (Narayanaswamy et al., 2009; Munder et al., 2016).

Because of the fast equilibration of ions between the cytosol and the medium after membrane permeabilization, it could be suggested that the incubation of irreversibly permeabilized cells in water contributes additionally to cytoplasmic proteins association and formation of different kinds of aggregates, which occurs naturally in stationary phase cells.

The yeast cell wall is an elastic, layered structure with a thickness of 100–200 nm (Klis et al., 2006). The outer mannoprotein layer limits the permeability of the wall for macromolecules and confers a negative charge to the cell surface. With the transition to stationary growth phase, the cell wall becomes thicker and less porous (Valentin et al., 1987; de Nobel et al., 1990).

Although the electrical treatment has some influence on the cell wall porosity it does not lead to yeast cells lysis (Ganeva et al., 2003, 2014). Therefore, the low extraction efficiency in water and in buffer with pH 5–6 could be attributed to the aggregation of the soluble cytoplasmic proteins, which prevents their diffusion through the wall.

Other groups working with spent brewer’s yeast and dry wine yeast (Liu et al., 2012, 2013) also observed a low protein extractability (2.78–4%) from electropermeabilized cells diluted in water. Their results, as well as data obtained in this study, are in discrepancy with the data reported earlier by Monch and Stute (2002) according to which, PEF treatment in a batch mode led to 30% release of proteins from baker’s yeast diluted in water. As no information is given about what is accepted in their study for 100% protein, the observed discrepancy could be due to differences in the protocol used for cell disruption and protein extraction (i.e., underestimation of the total protein content), or to the different methods used for protein quantification.

#### Influence of Cell Concentration

An important factor that can significantly modulate the effect of PEF on membrane integrity is the cell concentration. Recently, while studying the electroextraction of cytoplasmic enzymes from yeast, we found that there is no change of the electrical treatment efficiency at least up to cell concentration of 50 gDCW L^–1^, and also that the increase of the cell concentration led to reduction of the optimal field strength. We assumed that this effect is due to the strong conductivity increase during treatment of denser suspensions (Ganeva et al., 2003).

Here, we explore in more details the influence of the cell concentration on the efficiency of protein release. The suspensions used in these experiments had a biomass concentration corresponding to 12.5, 25, 37.5, 50, 66, 70, and 85 gDCW L^–1^. The pulsing chamber and the treatment conditions were the same as in the previous experiment. The only parameter varied to optimize the protein liberation was the electric field strength. After treatment, the cells were diluted in PPB pH 8.75 to final concentrations between 12.5 and 17 gDCW L^–1^, DTT was added to a final concentration of 2 mM and the suspensions were incubated for 16 h at room temperature.

**FIGURE 2:**
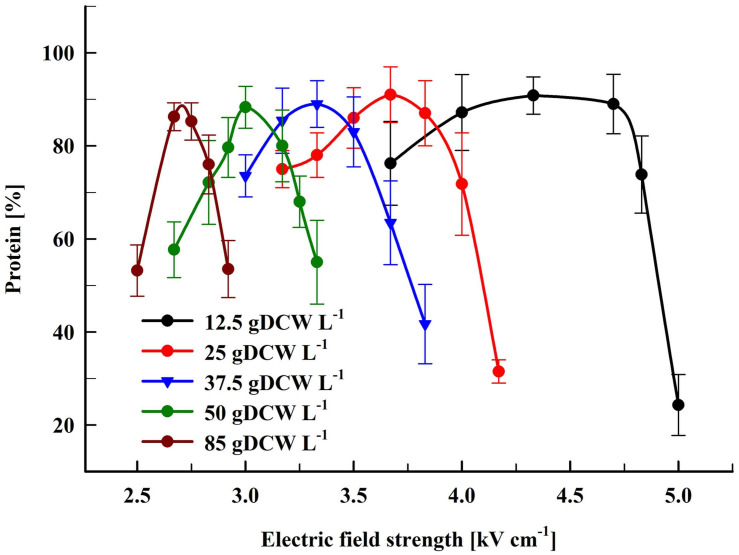
Influence of the field strength on the protein release efficiency. Cell suspensions with biomass concentration corresponding to 12.5–85 gDCW L^–1^ were treated with 15 pulses of 0.8 ms duration, frequency 7.5 Hz, flow rate 9 mL min^–1^. After PEF treatment, the cells were diluted in PPB pH 8.75 and incubated for 16 h at room temperature. 100% corresponds to the total protein in the cell lysate. The values represent the mean ± SD of three to six different experiments.

Figure 2 shows the dependence of the protein release on the field strength for five different concentrations. The maximal protein yield was between 86 and 91% from the total. This was achieved at field strengths where more (over 98%) of the cells were irreversibly permeabilized as detected by PI labeling. Treatment of the cells at field strength above the optimal led to a strong decrease in the protein extractability. A similar dependence on the field strength was observed for all tested concentrations. The specific treatment energy per liter of cell suspension (W_sus_) at optimal electrical conditions was in the range of 100–140 kJ L^–1^.

The reported in the literature data about the influence of cell concentration on PEF treatment efficiency are contradictory. This could be due to the different cell types utilized in these studies—mammalian cells or microorganisms (yeast, algae), the differences in conductivity of the electropermeabilization media, the different mode of PEF treatment—batch treatment, or treatment by using a continuous-flow chambers (Gášková et al., 1996; Álvarez et al., 2000; El Zakhem et al., 2006; Pucihar et al., 2007; Goettel et al., 2013).

The data we present here showed that there is no significant change of PEF efficiency at least up to yeast biomass concentration of 85 gDCW L^–1^. On the other hand, the increase of the concentration led to a significant reduction of the optimal field strength.

Studying the extraction of different intracellular components from algae, Goettel et al. (2013) found, that the PEF efficiency is not affected even at biomass concentrations of 167 gDCW L^–1^. The authors reported a significant reduction of the PEF-demand energy (MJ kgDCW^–1^) with the increase of the biomass concentration.

In our experiments, the increase of the yeast cell concentration from 12.5 to 85 gDCW L^–1^ resulted in PEF energy reduction from 10.5 to about 1.56 MJ kgDCW^–1^. Despite the applied different electrical conditions to induce irreversible permeabilization—i.e., moderate field strength (2.5–5 kV cm^–1^) and series of relatively long (0.8 ms) pulses, the PEF-demand energy required for maximal protein recovery from yeast was close to the one reported about algae suspensions with similar concentration. Based on the results obtained by Goettel et al. (2013), it seems possible to increase further the yeast biomass without changing the PEF treatment efficiency.

#### Upscaling of the PEF Treatment Protocol: Temperature Effect on the Protein Release Efficiency

PEF treatment in a flow mode can be easily scaled up by preserving the same extraction efficiency. For example, an increase in the flow rate can be obtained by using pulsing chambers with larger volumes and by pulse frequency increase.

**FIGURE 3:**
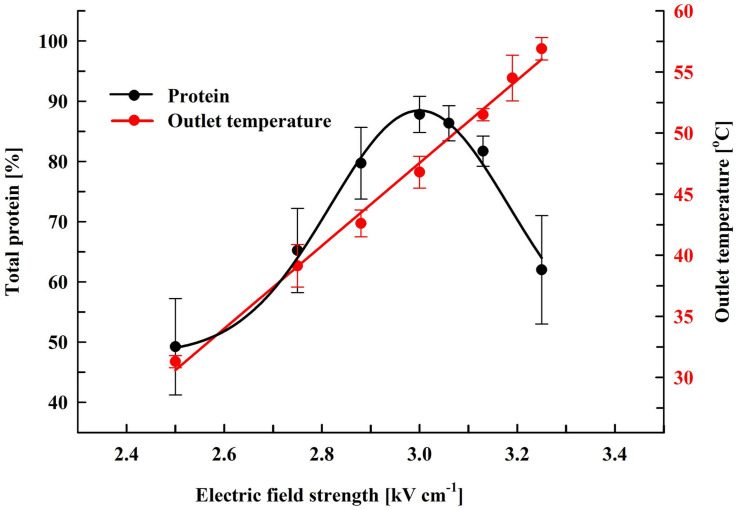
Influence of field strength on protein release efficiency and outlet temperature. Electrical conditions: 19 pulses of 0.5 ms duration, pulse frequency 17.1 Hz, flow rate 27 mL min^–1^. After PEF treatment, the cells were diluted threefold in 250 mM PPB pH 8.75 containing 2 mM DTT and incubated for 16 h at room temperature. 100% corresponds to the total protein in the cell lysate. The values represent the mean ± SD of five different experiments.

Here, we performed experiments on protein extraction by using a chamber with a volume of 0.5 mL. Firstly, the cell suspension (66 gDCW L^–1^) was treated at flow rate of 27 mL min^–1^. During their passage through the chamber the cells received 19 pulses (pulse frequency 17.1 Hz) of 0.5 ms duration which corresponds to a total treatment time of 9.5 ms. After electrical treatment, the suspensions were diluted in PPB containing DTT and then incubated at room temperature for 16 h. At these conditions, maximal protein release (87.8 ± 3%) was obtained at field strength of about 3 kV cm^–1^ (Figure 3). The specific treatment energy per liter of cell suspension (W_sus_) was 120 kJ L^–1^. As already shown (Figure 2), treatment at higher field strengths led to a significant reduction in the protein yield.

Next, we studied the protein extraction efficiency at higher flow rate—130 mL min^–1^. To assure that the cells receive the same pulse number (19 pulses, total treatment time of 9.5 ms), the pulse frequency was increased to 82.3 Hz and the PEF treatment optimization was performed only by varying the electric field strength. The permeabilized cells were diluted and incubated at room temperature as already described. We found that at this flow rate the same extraction efficiency is reached at field strength in the range of 2.75–2.88 kV cm^–1^ (Supplementary Material 2). At 3 kV cm^–1^ the protein yield started to decrease and at 3.16 kV cm^–1^ the difference between the protein yields obtained at 27 and 130 mL min^–1^ became statistically significant (*P* < 0.01).

Although the PEF treatment is considered as a non-thermal method, it is associated with a temperature rise due to Joule heating. This effect could be significant when dense cell suspensions are treated under conditions leading to irreversible permeabilization, such as in our experiments. The observed phenomenon is consequence from the strong increase in conductivity during pulse application because of an instantaneous release of intracellular ions. We measured the outlet temperature at the different field strengths applied. At a flow rate of 27 mL min^–1^, in our system the residence time of the cells inside the treatment chamber was about 1.1 s and the outlet temperature was registered about 4 s after the cells received 19 pulses with 0.5 ms duration. We found that the outlet temperature reached a maximal value about 20 s after the start of the pulsation and then remained stable despite the lack of cooling system. As shown in Figure 3, at field strengths leading to maximal protein yields, the outlet temperature was in the range of 46–49°C.

Treatment of the suspensions at about 3.13 kV cm^–1^ (outlet temperatures over 50°C) led to a significant (*P* < 0.01) decrease in the protein release and at about 3.25 kV cm^–1^ (outlet temperature of 56–58°C) the yield dropped to 62 ± 9%. We assumed that the decrease observed might be a result of temperature-induced change in the conformation of the cell wall mannoproteins. Changes in the conformation of the soluble intracellular proteins could further complicate their diffusion through the wall.

**FIGURE 4:**
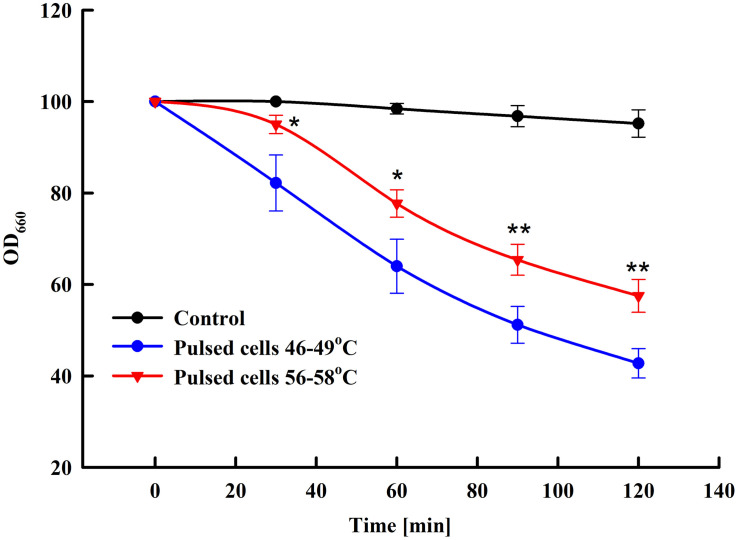
Influence of PEF treatment on lyticase sensitivity of baker’s yeast. Electrical conditions: 19 pulses of 0.5 ms duration, pulse frequency 17.1 Hz, flow rate 27 mL min^–1^, field strength 3 kV cm^–1^ (outlet temperature 46–49°C) and 3.25 kV cm^–1^ (outlet temperature 56–58°C). Electrically treated and control cells were diluted in PPB pH 7.5, lyticase was added to final concentration 60 U mL^–1^ and the cells were incubated at 30°C. The values represent the mean ± SD of three different experiments. *P < 0.05; **P < 0.01.

Yeast susceptibility to lytic enzymes is a routine approach used to reveal changes in the structure of the outer mannoprotein layer of the yeast cell wall under influence of different factors (de Nobel et al., 2000; Simões et al., 2003; Ganeva et al., 2014). We compared the sensitivity to lytic enzyme of control cells and cells treated at two different field strengths: 3 and 3.25 kV cm^–1^. As illustrated in Figure 4, at optimal field strength there is a significant increase of cell sensitivity to lytic enzyme, while treatment at higher field strength led to a statistically significant reversal of the effect and respective decrease of the cell sensitivity to lyticase.

To check for temperature-induced changes in the conformation of the soluble proteins, we compared the activity of the enzyme alcohol dehydrogenase (ADH) in the lysates prepared from control cells and cells treated at two different field strengths—3 kV cm^–1^ (outlet temperature 49°C) and 3.25 kV cm^–1^ (outlet temperature 57–58°C). We found that cell treatment at 3 kV cm^–1^ (optimal conditions for total protein extraction) did not affect the ADH activity. On other hand, ADH activity of cells pulsed at 3.25 kV cm^–1^ was 20 ± 1.8% lower than this of the control, untreated cells. It has to be pointed out that in our experiments the temperature of the collected samples incubated at room temperature decreased very fast and in a few minutes (3–5 min) after electrical treatment it reached values of 40–45°C.

Based on the results from the lyticase test and ADH activity measurement, we concluded that at electrical conditions leading to maximal protein extraction, no significant protein denaturation takes place. However, these results also showed that under certain electrical conditions the temperature rise, which occurred during pulse application, could provoke some conformational changes in both, the cell wall mannoproteins and the soluble proteins, causing reduced diffusion efficiency through the cell wall.

There are numerous data obtained with different cell types indicating that the temperature of the samples (cell suspension or tissue) during pulse application influences electropermeabilization. Experiments with algae, mammalian cells and animal tissues demonstrated that electrical treatment at physiological temperatures is more efficient than treatment of samples chilled to 4–5°C (Coster and Zimmermann, 1975; Gallo et al., 2002; Kandušer et al., 2008).

There is also evidence, that moderate preheating of the samples improves electropermeabilization and lowers the energy consumption. Synergism between thermal and electric field effect has been observed for PEF microorganism inactivation (Wouters et al., 1999; Heinz et al., 2003; Amiali et al., 2007) and electrically induced extraction of intracellular compounds from plant tissues and algae (Lebovka et al., 2005; Postma et al., 2016). One of the possible reasons for these effects seems to be the temperature-induced increase of membrane fluidity, which facilitates the electroinduced loss of plasma membrane integrity (Kandušer et al., 2008).

In our experiments, the inlet temperature was about 24°C and the temperature rise of the suspensions at optimal electrical conditions was 22–25°C. Since we do not control the temperature during the pulsation, it is difficult to separate the contribution of the PEF effect and the thermal effect, but it seems highly probable, that the temperature increase during pulse application facilitates electropermeabilization. On the other hand, as mentioned above, higher temperature increase has an inverse effect on the protein release, which could be explained by temperature-induced changes in the conformation of the cell wall mannoproteins and the cytoplasmic proteins.

Recently, working with the thermotolerant yeast *Hansenula polymorpha*, we obtained maximal protein extraction at electrical conditions where outlet temperatures of 59–60°C had been reached (Ganeva et al., 2018). It could be assumed that the effect of the temperature on the protein release efficiency depends on the system particularity—the cell wall composition and structure, the plasma membrane composition, the thermostability of the soluble proteins.

**FIGURE 5:**
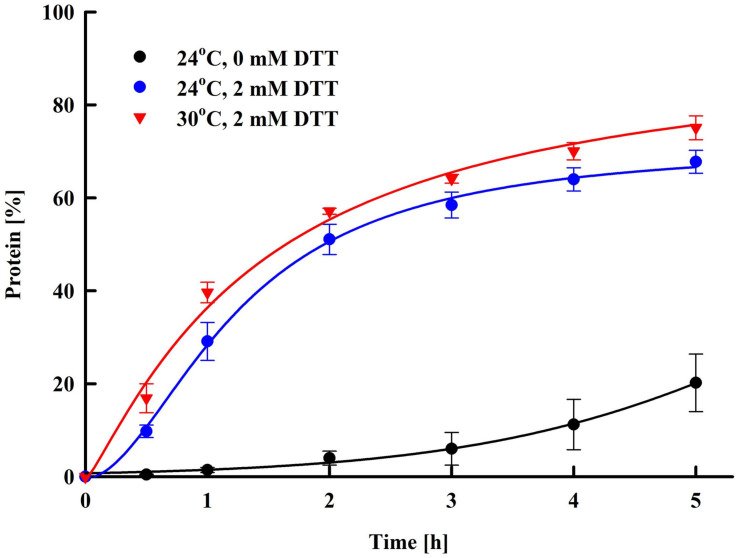
Electroinduced release of total protein at various periods after PEF treatment. Electrical conditions: 19 pulses of 0.5 ms duration, pulse frequency 17.1 Hz, flow rate 27 mL min^–1^, field strength 3 kV cm^–1^. After PEF treatment, the cells were diluted in 250 mM PPB pH 8.75 with or w/o 2 mM DTT and incubated at room temperature or at 30°C. 100% corresponds to the total protein in the cell lysate. The values represent the mean ± SD of three different experiments.

In our experiments, high protein yield was achieved by incubation of the pulsed cells at room temperature, which in terms of performance is simple and cost-effective. On the other hand, the prolonged incubation (16 h) carries some risks of bacterial contamination. We evaluated the protein release rate and found that main release (68.6 ± 5.4%) occurred during the first 5 h of post-pulse incubation (Figure 5). The thiol compound, which increases cell wall porosity by reducing the disulfide bonds between the cell wall mannoproteins (Zlotnik et al., 1984), significantly enhanced the yield. As diffusion is a temperature-dependent process, we tried to accelerate the protein release by incubation of the cells at a higher temperature, as done earlier for the extraction of intracellular proteins (Ganeva et al., 2003, 2018). The increase of the temperature from 24 to 30°C had some impact on the rate and efficiency of protein release mainly during the first several hours, however, the maximal yield reached after 14–16 h of incubation at these two temperature was rather similar. These results led to the conclusion that the incubation of the permeabilized cells can be performed at room temperature without significant decrease of the protein yield.

### Nucleic Acid Content of the Extracts

Once the proteins are extracted, they have to be concentrated and purified to remove the undesired or potentially harmful substances coming from the cells or introduced during extraction (Middelberg, 2012). Ultrafiltration is a commonly employed method for protein concentration, buffer exchange and purification in the biotechnology industry (van Reis and Zydney, 2007). That is why we applied this technique to concentrate the proteins released from the permeabilized cells. The electrical treatment and the conditions of post-pulse incubation are as described in the text to Figure 3. After 16 h of incubation at room temperature the cells were removed by centrifugation and the supernatants obtained were subjected to ultrafiltration at 10 kDa.

Next, after appropriate dilution the protein and NA content in the retentates were determined. The percentage decrease of the protein content after ultrafiltration was 3 ± 0.2%, which most probably was due to some protein retention by the membrane as we did not detect protein in the eluate. This result suggests that at the applied conditions (alkaline pH, room temperature) no significant proteolytic degradation occurred despite of the prolonged time of incubation. On the other hand, the protein/NA ratio in the concentrated supernatants was 100/5.6 ± 0.9.

As shown in Figure 5, the permeabilized cells diluted in buffer without DTT released only about 1.6–2.2% protein during the first 1–1.5 h incubation. Therefore, we evaluated also the amount of released NA by using a different protocol for post-pulse incubation. In these experiments a pretreatment step was introduced, where after electrical treatment, the cells were diluted in PPB without DTT and incubated for 1.5 h. Afterward, the supernatant was removed, the cells were diluted in PPB with DTT and incubated as described above. We found that this pretreatment of the cells led to an additional decrease of the protein/NA ration −100/4.5 ± 0.3.

The main limiting factor for utilization of yeast biomass as a protein source in human diet is the high NA content, because in human, the purine nucleotides are metabolized to uric acid. Reduction of the purine content of yeast has been studied extensively for the past few years and different methods based on chemical or enzymatic treatment have been developed (Halász and Lásztity, 1991; Nasseri et al., 2011). Although they allow production of protein concentrates with low NA and nucleotides content, several problems associated with their application exist. Chemical processing, often applied in combination with heating, can result in alteration of the amino acid profile, to formation of potentially toxic products, such as lysinoalanine and to denaturation, which decreases the nutritive properties of the proteins (Shetty and Kinsella, 1979; Halász and Lásztity, 1991; Nasseri et al., 2011). The enzymatic methods based on the utilization of exogenous nucleases are limited because of the cost of the catalyst, while NA reduction by autolytic degradation is often performed under conditions where protein hydrolysis also occurs (Trevelyan, 1976).

According to the literature, the protein/NA ratio for the lysates obtained by mechanical disintegration of *S. cerevisiae* is in the range of 100/12–100/25 (Shetty and Kinsella, 1979; Halász and Lásztity, 1991). The lower NA content in the protein concentrates obtained after PEF treatment can be explained by the fact, that about 80% of the yeast RNA is ribosomal (Waldron and Lacroute, 1975; Warner, 1999) and is part of large nucleoprotein complexes, which generally cannot be released from the permeabilized cells. As there is no cell lysis, the DNA is also retained inside, and the NA released after plasma membrane permeabilization are most probably mainly tRNAs and mRNAs.

Depending on the yeast type and growth conditions, the protein content of the fresh baker’s yeast ranges between 40.8 and 58% of the dry cell matter (Bekatorou et al., 2006). According to Kinsella (1987) only 40% from the total protein in yeast is soluble (cytoplasmic) protein, the other part consists of ribosomal protein (40%) and cell wall protein (20%). The protein yield we obtained after disruption of the cells with glass beads was 199 mg gDCW^–1^, which corresponds approximately to the average content of soluble protein in baker’s yeast.

The data presented in this study demonstrated that the PEF treatment applied in flow mode, followed by incubation of the electropermeabilized yeast cells in a buffer with alkaline pH, resulted in a highly efficient extraction of the intracellular soluble protein with low content of NA. The protein yield of 170–180 mg gDCW^–1^ is lower than that obtained by high pressure homogenization (Sergeev et al., 1984; Jacob et al., 2019). On the other hand, PEF treatment is a milder technique, which does not provoke protein denaturation when used with the appropriate combination of electrical parameters. The release of intracellular compounds, including proteins, is a result of plasma membrane permeabilization and there is no cell fragmentation, which allows separation of the cells by microfiltration or low speed centrifugation. The release of the proteins is a slow process, but the incubation of the permeabilized cells in alkaline pH buffer and temperatures of 24–30°C seems to prevent the protein hydrolysis. There are data indicating that electrical treatment performed in a batch and continuous mode caused accelerated yeast autolysis. It has to be pointed out that in these studies the treated cells were incubated for several days at 18–26°C (Martinez et al., 2016; Maza et al., 2020) or several hours at 40–60°C and pH 6.6 (Monch and Stute, 2002).

The results from our study demonstrated the principal possibility for utilization of PEF treatment for production of protein concentrates with low NA content, thus suitable for human consumption. The proteins were not modified due to thermal or chemical treatment during the extraction process. The reduction of the NA content did not require additional chemical or enzymatic treatment, while the concentration by ultrafiltration also allowed the removal of the nucleotides liberated during post pulse incubation. We do not exclude, that some changes of post-pulse incubation, as well as utilization of membranes with a cutoff higher than 10 kDa could lead to further reduction of the NA without substantial loss of proteins.

Since the treated cells become more sensitive to lytic enzymes (Ganeva et al., 2014, 2018) and to mechanical disintegration (Shynkaryk et al., 2009), the residual biomass after removal of the protein, could be further processed to induce autolysis (Monch and Stute, 2002; Maza et al., 2020) or it could be disintegrated by high pressure homogenization for recovery of additional valuable compounds—amino acids, nucleotides, proteins, and polysaccharides.

### Electroinduced Extraction of Low Molecular Compounds

As shown in this study, incubation of the electropermeabilized baker’s yeast in water led to very poor protein release, which is most probably a result of protein aggregation and retention by the cell wall. These incubation conditions, however, do not prevent the diffusion of small water-soluble substances through the yeast cell wall (Monch and Stute, 2002; Shynkaryk et al., 2009).

We evaluated the rate and efficiency of liberation of different intracellular compounds—free amino acids, antioxidants and nucleotides, from irreversibly permeabilized cells. In these experiments, we applied the electrical conditions, which we found as optimal for protein extraction (Figure 3). Cell suspensions (66 gDCW L^–1^) with conductivity of 150 μS cm^–1^ were treated at a flow rate of 27 mL min^–1^ in pulsing chamber with 0.5 mL volume with 19 pulses of 0.5 ms duration (total treatment time of 9.5 ms). The field strength was set at 3 kV cm^–1^. After pulsation, the suspensions were incubated without dilution at room temperature. Samples were taken at defined time intervals, the cells were removed by centrifugation and the supernatants were analyzed for the different substances. As 100% was set the content of the analyzed compound in the soluble fraction obtained after mechanical disintegration of suspensions from control untreated cells with the same concentration.

**FIGURE 6:**
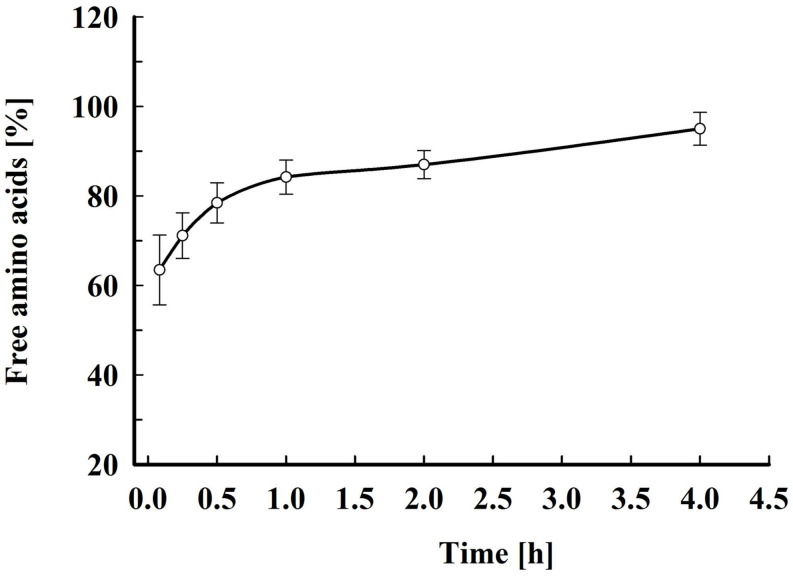
Electroinduced release of free amino acids at various periods after PEF treatment. Electrical conditions: as described in Figure 3. After PEF treatment, the cell suspensions were incubated at room temperature without dilution. 100% corresponds to the free amino acid content in the cell lysate. The values represent the mean ± SD of five different experiments.

As shown in Figure 6, about 70% of the free amino acids were released 15 min after electrical treatment. Afterward, the amino acids leakage continued slowly and at 4 h after pulsation the yield reached 90–95% from total content corresponding to 51.8 ± 8.6 mg amino acids gDCW^–1^. Taking into account that the free amino acids are distributed between the cytosol and the vacuole (Kitamoto et al., 1988), it could be hypothesized that there is also a change in the vacuolar membrane’s integrity. Vacuoles in stationary phase cells are large organelles, which occupy most of the intracellular space, and it is possible that in part of the cells the loss of vacuolar membrane integrity is a direct effect of the electrical treatment (i.e., electropermeabilization). However, given that the release of the free amino acids, which are small molecules, continues for several hours, it is possible that the subsequent vacuolar rupture is caused by osmotic imbalance.

Yeast antioxidant potential is determined by different soluble and insoluble cell components (Santiago and Mori, 1993; Jaehrig et al., 2008). According to Santiago and Mori (1993), 85–90% of the free radical scavenging activity of the soluble fraction obtained after mechanical cell disruption is due to thermostable compounds with molecular mass under 10 kDa. Yeast have different low molecular compounds with antioxidant properties but one of the most important antioxidants in the cell is glutathione. *S. cerevisiae* and *Candida utilis* are currently used for industrial production of glutathione, which has found application in pharmaceutics, as an additive for the production of functional foods and as a natural antioxidant for preservation of food quality (Li et al., 2004). To assess the applicability of PEF treatment for extraction of antioxidants from yeast we determined the total antioxidant activity, the glutathione and the total phenolic metabolites released from the cells, at different time intervals after pulsation. As in previous experiments, the cell suspensions were incubated at room temperature without dilutions.

**FIGURE 7:**
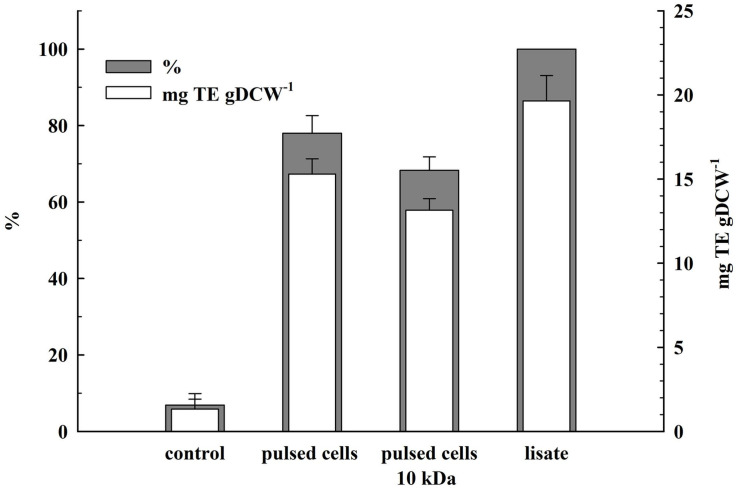
Release of total antioxidant activity from electrically treated and control cells. Electrical conditions: as described in Figure 3. Control and electrically treated cells were incubated at room temperature for 2 h without dilution. 100% corresponds to the total antioxidant activity in the cell lysate. The values represent the mean ± SD of three different experiments.

The total antioxidant activity in the lysate and in the supernatants obtained 2 h after PEF treatment was determined by estimating the ABTS radical-scavenging activity. As shown in Figure 7, the permeabilized cells released 78 ± 4.6% of the total antioxidant activity corresponding to 19.65 ± 1.5 mgTE gDCW^–1^. The antioxidant activity of the permeate obtained after ultrafiltration of the supernatants at 10 kDa was 68.3 ± 3.5% which brought us to the conclusion that the antioxidant activity released after electrical treatment and incubation of the cells in water is mainly due to low molecular compounds.

We evaluated the glutathione liberation, as well. The release of this cytosol tripeptide was almost instantaneous—10 min after pulsation 78 ± 6% from the total content corresponding to 9.6 ± 0.5 mg gDCW^–1^ was detected in the supernatant of the permeabilized cells. Further incubation (up to 4 h after pulsation) did not improved the yield.

**FIGURE 8:**
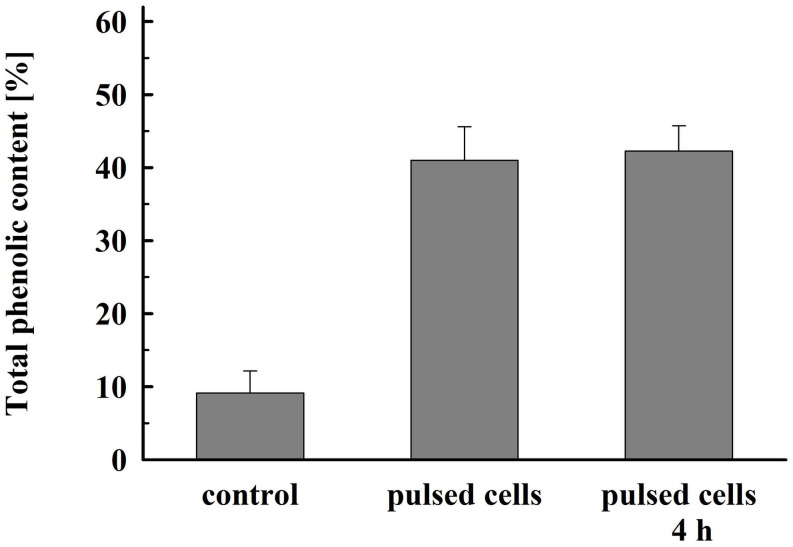
Total phenolic content released from electrically treated and control cells. Electrical conditions: as described in Figure 3. Control cell were incubated for 4 h, electrically treated cells were incubated for 1 h and, respectively, 4 h. 100% corresponds to the total phenolic content in the cell lysate. The values represent the mean ± SD of four different experiments.

As illustrated in Figure 8, permeabilized cells released about 41% of their total phenolic content 1 h after pulsation. Prolonged incubation did not significantly improve the yield. This lower extraction efficiency could be due to specificities in their intracellular location and/or interaction with other cellular components such as cell wall (Mohamed et al., 2018).

**FIGURE 9:**
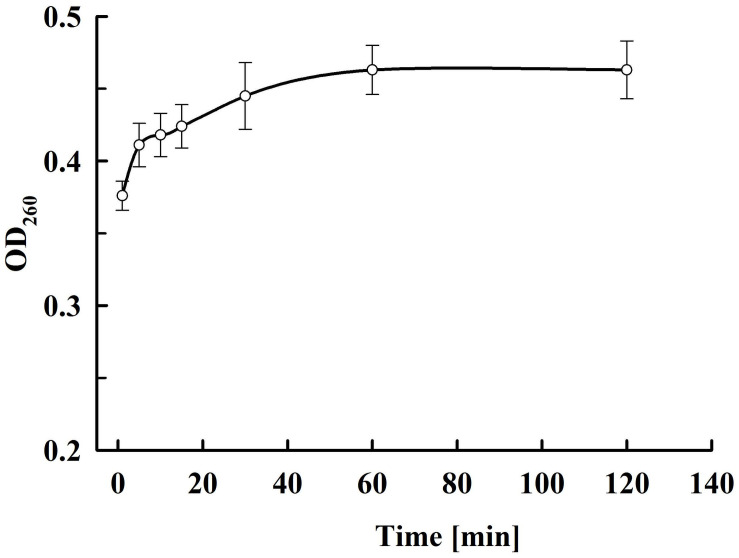
Release of UV absorbing substances at various periods after PEF treatment. Electrical conditions: as described in Figure 3. The values represent the mean ± SD of four different experiments.

As shown in Figure 9, there is very rapid release from the electropermeabilized cells of substances absorbing at 260 nm. Maximal absorbance was observed about 60 min after electrical treatment. No further changes were registered even after prolonged (up to 20 h) incubation of the electropermeabilized cells at room temperature. Therefore, we assumed that the absorbance was mainly due to leakage of nucleotides, nucleosides, cofactors, and other low molecular weight compounds. We measured the absorbance of the permeate obtained after ultrafiltration of the supernatants at 10 kDa cut off and found only 7.2 ± 1% decrease which supported our assumptions. In addition, we measured the absorbance of the permeate obtained after ultrafiltration of the soluble part of the lysate and found that the electropermeabilized cells incubated in water release about 92% from the low molecular UV absorbing substances present in the cell. These data led us to conclude that when the permeabilized cells are incubated in water, RNA is generally retained inside, as shown for proteins. On the other hand, it seems that at the used incubation conditions yeast RNAses have very low (or even no) activity, otherwise we should have registered an increase in the absorption at 260 nm over time.

We also determined the total purine content of the supernatants obtained after electrical treatment and incubation of the cell suspension for 4 h at room temperature. The obtained value, calculated by gram dry biomass, was 2.13 ± 0.4 mg gDCW^–1^, which is significantly lower than the purine content of the whole cell (Kaneko et al., 2014).

The data presented here demonstrated that incubation of permeabilized cells in water led to liberation mainly of relatively low molecular compounds. At these incubation conditions, the soluble proteins and NA were largely retained inside. On the other hand, water extracts contained most (about 80%) of the total antioxidant activity and glutathione content of the cells. The cell suspensions used in this study were relatively dense—approximately 260 g fresh weight L^–1^. It is possible that the yield could be improved by re-extraction with water or by additional dilution.

It seems highly probable that other bioactive compounds localized in the cytosol, for example water soluble B-complex vitamins and small peptides, could be released with similar efficiency. The release of the low molecular compounds is fast, and there is no need for incubation at high temperature, or addition of chemicals (salt, solvents) or enzymes.

Our data demonstrate that the amino acid content of the studied extracts is significantly lower than what is reported for the autolysates (Jacob et al., 2019). This could be explained by the observation that PEF treatment itself does not provoke protein degradation and the following incubation of the permeabilized cells in water does not cause significant protein hydrolysis. Most importantly, it seems that at these conditions, there is also no NA degradation and, as a result, electropermeabilization led to leakage mainly of the free nucleotides present in the cells.

The main limitation for using whole yeast as a source of vitamins, antioxidants and other biologically active substances in human diet is the high NA content. The same problem exists with the utilization of autolysates (often indicated as yeast extracts) as a food additive, because they have practically the same purine content as intact cells. The water extracts obtained after PEF treatment have a very low level of purine nucleotides in comparison to whole cells. Thus, it is possible that after appropriate concentration they could be utilized for health supplements in human diet or as a source of glutathione for preservation of different food from oxidative damage without the risk of undesirable purine content increase.

PEF treatment by using continuous-flow chambers has all the prerequisites to be developed as an alternative method for extraction of glutathione and other low molecular compounds from yeast. The technique is suitable for large-scale biomass processing. Irreversible permeabilization of water suspensions with high biomass content can be achieved after a single pass through the pulsing chamber and the treatment can be performed at room temperature without using cooling system. After appropriate time of incubation, the pulsed cells, which have been demonstrated to have a strong tendency to aggregate (Shynkaryk et al., 2009), can be easily separated from the supernatants containing the low molecular bioactive compounds. Then these cells, which practically retain most of the macromolecules—proteins, polysaccharides, and NA, can be further processed for recovery of these valuable compounds or simply dried and distributed as an animal feed or fertilizers.

## Conclusion

The present study demonstrated that the PEF treatment applied in flow mode, followed by incubation of the electropermeabilized yeast cells in a buffer with alkaline pH, results in highly efficient extraction of the intracellular soluble proteins. At electrical conditions leading to maximal protein extraction, no significant protein denaturation takes place. The PEF energy needed to achieve irreversible electropermeabilization was significantly reduced with the increase of the biomass concentration. The protein release is a slow process, but the incubation at alkaline pH and room temperature seems to prevent the protein hydrolysis. The protein concentrates obtained by ultrafiltration had lower NA content in comparison to the cell lysates obtained by mechanical disintegration, most probably due to the retention of part of the NA (ribosomal RNA and DNA) inside the cells. The possibility for utilization of PEF treatment in a continuous flow mode for production of protein concentrates suitable for human consumption is suggested.

Incubation of permeabilized yeast cells in water led to leakage mainly of relatively low molecular compounds. The water extracts contain most of the total antioxidant activity, glutathione and phenolic compounds present in the cell, and in same time had a very low level of purine nucleotides in comparison to whole cells. These specificities in their composition make the yeast water extracts suitable for utilization as supplements representing a source of natural antioxidants for health purposes and in the food industry.

## Data Availability Statement

The original contributions presented in the study are included in the article/Supplementary Materials, further inquiries can be directed to the corresponding author.

## Author Contributions

VGa and BG designed the study. VGa, BA, and MZ contributed to data acquisition and read and approved the final version of the manuscript. VGa, BG, and VGo performed the data analysis and data interpretation. VGa wrote the manuscript. All authors contributed to the article and approved the submitted version.

## Conflict of Interest

The authors declare that the research was conducted in the absence of any commercial or financial relationships that could be construed as a potential conflict of interest.

## Supplementary Material

Supplementary Material 1 Schematic representation of the experimental setup used for PEF treatment.

Supplementary Material 2 Influence of field strength on protein release efficiency. Electrical conditions: 19 pulses of 0.5 ms duration, flow rate 27 mL min^–1^ (pulse frequency 17.1), flow rate 130 mL min^–1^ 82.3 Hz. Hz, flow rate. After PEF treatment, the cells were diluted threefold in 250 mM PPB pH 8.75 containing 2 mM DTT and incubated for 16 h at room temperature. 100% corresponds to the total protein in the cell lysate. The values represent the mean ± SD of four different experiments (***P* < 0.01).
