# Supplementary figures and images for: Extraction of Proteins and Other Intracellular Bioactive Compounds From Baker’s Yeasts by Pulsed Electric Field Treatment
Source: Front Bioeng Biotechnol. 2020 Dec 15;8:552335. doi: 10.3389/fbioe.2020.552335 (PMC7770146; doi:10.3389/fbioe.2020.552335)

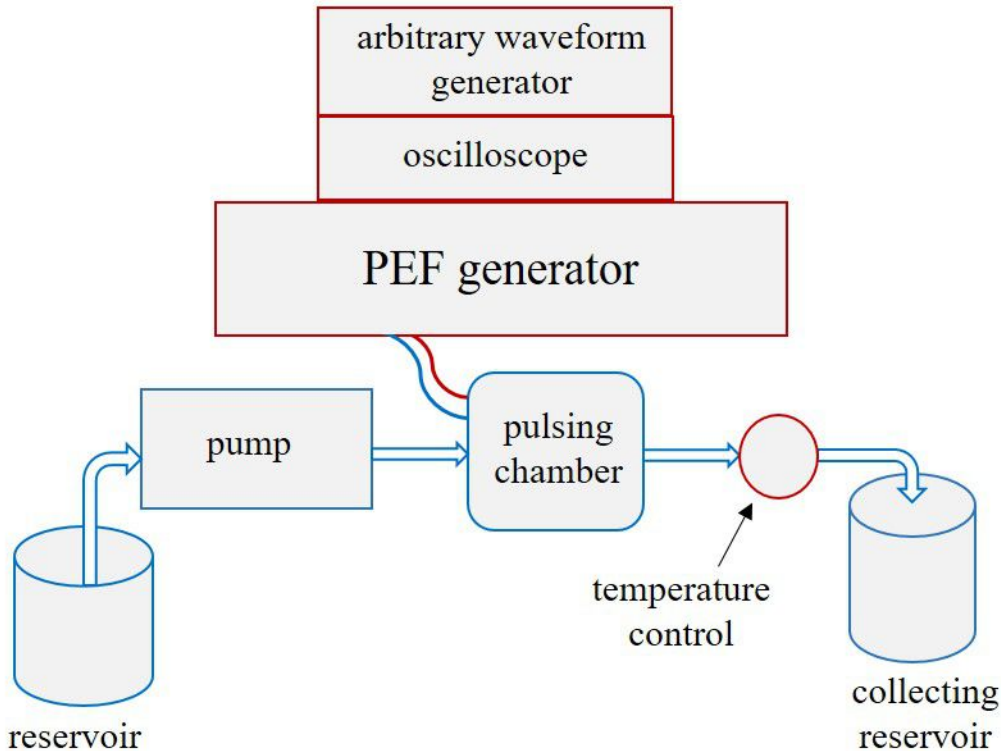

Supplement: Supplementary Material 1 — Schematic representation of the experimental setup used for PEF treatment. [file Image_1.pdf]
